# Pathogenic Characterization and Host Immune Response to *Vibrio harveyi* in Diseased *Seriola dumerili*

**DOI:** 10.3390/ani16020184

**Published:** 2026-01-08

**Authors:** Rizhao Zhang, Jingbo Hu, Xin Zhao, Kunpeng Lan, Haimin Tan, Yang Huang, Chunhua Zhu

**Affiliations:** 1 Fisheries College, Guangdong Ocean University, Zhanjiang 524088, China; zhangriz@163.com (R.Z.); 18077903787@163.com (H.T.); zjouhy@126.com (Y.H.); 2 Development and Research Center for Biological Marine Resources, Southern Marine Science and Engineering Guangdong Laboratory (Zhanjiang), Zhanjiang 524006, China; hjb574660391@gmail.com (J.H.); 15505926918@163.com (K.L.); 3 Guangdong Research Center on Reproductive Control and Breeding Technology of Indigenous Valuable Fish Species, Guangdong Ocean University, Zhanjiang 524088, China; 4 Guangdong Provincial Engineering Laboratory for Mariculture Organism Breeding, Zhanjiang 524088, China

**Keywords:** *Seriola dumerili*, *Vibrio harveyi*, bacterial pathogenesis, transcriptome, immune response

## Abstract

The greater amberjack (*Seriola dumerili*) is an economically important marine species that is prone to bacterial infections, resulting in high mortality rates and substantial economic losses. In this study, a virulent bacterial strain, Vh-2, was isolated from diseased greater amberjack and identified as Vibrio harveyi. Experimental infections caused high mortality and severe splenic damage characterized by tissue necrosis, abnormal pigment deposition, cellular disintegration, and extensive immune cell infiltration. A virulence gene analysis revealed that Vh-2 harbored multiple virulence-associated genes such as *toxR*, *toxS*, *vhpA*, *vhpB*, *vhhA*, *vhhB*, *luxR*, and *pap6*. Antibiotic susceptibility testing demonstrated ampicillin resistance but sensitivity to ceftriaxone, florfenicol, and meropenem. Transcriptomic profiling of infected spleens identified 396 differentially expressed genes (DEGs) compared to the control group, of which 293 were upregulated and 103 were downregulated. A functional enrichment analysis indicated that these genes were primarily involved in cell cycle regulation, DNA repair, metabolic processes, and immune-related pathways. These findings enhance our understanding of *V. harveyi* pathogenesis and immune responses of *S. dumerili* and provide new insights into the prevention and control of *V. harveyi* infections in marine fish.

## Simple Summary

The greater amberjack (*Seriola dumerili*) is an important farmed fish that often becomes sick from harmful bacteria, causing serious losses in aquaculture. In this study, we examined a disease-causing bacterium taken from sick fish and confirmed that it can trigger illness in healthy fish. We also studied how the fish reacts after infection by looking at changes in gene activity in its immune tissues. The results show that many genes related to defense and inflammation become more active. These findings help us better understand how the fish fights infection and may support improved disease control in aquaculture.

## 1. Introduction

*Vibrio* spp. are known to infect aquatic animals, resulting in significant economic losses to the aquaculture industry [1]. Among these, *V. harveyi* is a Gram-negative, halophilic, facultative anaerobic bacterium commonly found in warm marine environments and is opportunistically pathogenic in fish and crustaceans. Early symptoms of *V. harveyi* infection in marine fish include lethargy, reduced feeding, abnormal swimming behavior, and skin ulceration, occasionally accompanied by tail rot, exophthalmia, and abdominal distension or ascites [2,3]. In severe cases, *V. harveyi* can cause splenic necrosis, liver inflammation, and bacterial invasion of internal organs. In *V. harveyi*-infected gilthead seabream (*Sparus aurata*), hemosiderin deposits and melanomacrophage centers have been observed in the spleen [4]. *Vibrio harveyi* demonstrates significant pathogenic diversity as strains differ in their virulence gene profiles. It harbors key virulence genes, including *rtxD*, *acfD*, *toxR*, *luxS*, iron acquisition-related genes *(sitA*, *sitB*, *sitC*, and *sitD*), and the heat-stable cytotoxic enterotoxin gene ast [5]. A pathogenic strain isolated from diseased fish possessed the conserved regulator *toxR* and its deletion resulted in increased biofilm formation, highlighting its crucial role in colonization and virulence [6].

Transcriptome analysis, a commonly used approach to assess host–pathogen interaction mechanisms, effectively reveals genes involved in immune responses [7,8]. A comparative transcriptomic study of Japanese pufferfish (*Takifugu rubripes*) revealed differential gene expression profiles in the liver and spleen following *V. harveyi* infection, highlighting the activation of multiple immune-related pathways [9]. Similarly, a transcriptome analysis identified a broad range of immune genes involved in the response of spotted sea bass (*Lateolabrax maculatus*) to *Edwardsiella tarda* infection [10]. Compared to the conventional digital gene expression (DGE) analysis, the RNA-Seq approach is significantly advantageous as it can detect low-abundance transcripts, identify variable splicing events, and reveal novel genes [11]. Because of the efficiency of transcriptome analysis in identifying immune markers, it establishes a theoretical foundation for the optimization of aquaculture disease prevention and control. The fish spleen serves as a primary immune organ rich in lymphocytes, macrophages, and granulocytes, and is essential for innate and adaptive immunity, such as cytokine production and antigen presentation [12,13]. Its primary function in bacterial resistance highlights the necessity of transcriptome analysis to explore spleen immune mechanisms and provide a molecular basis for disease prevention.

The greater amberjack (*Seriola dumerili*) is a warm-water marine fish globally distributed in tropical and subtropical seas [14]. Due to its rapid growth rate, high quality meat, and adaptability to net-pen aquaculture habitats, it has become an economically important aquaculture species [15]. Over the past decade, aquaculture production of greater amberjack has increased significantly due to an increased consumer demand for high-quality products and the continuous increase in its market value [16]. However, increased stocking density and pollution have caused regular outbreaks of various bacterial diseases, which have considerably impacted aquaculture development [17,18].

The pathogenesis of *V. harveyi* and the immune response of greater amberjack to infection are not well known. Therefore, this study aims to investigate the pathogenic mechanisms of this bacterium isolated from diseased *S. dumerili* by bacterial culture, morphological observation, and 16S rDNA gene sequencing, and explore the host immune response mechanisms through transcriptomic analysis of the spleen. This study will provide new insights into the prevention and control of *V. harveyi* infection in aquaculture farms.

## 2. Materials and Methods

### 2.1. Fish Collection and Inspection

The moribund greater amberjacks were collected from the aquaculture farm on Donghai Island, Zhanjiang, China. A microscopic examination was conducted on fresh gill filaments, mucus, and internal organs (liver, spleen, kidney, and intestine) to detect the presence of parasites.

Healthy greater amberjacks (*n* = 20) from 55–60 cm in length and 2000–2800 g in weight were attained from the aquaculture farm on Donghai Island. The fish were transported to the Guangdong Southern Marine Science and Engineering Laboratory (Zhanjiang, China) for the artificial infection analyses.

### 2.2. Isolation and Identification of Bacteria

Moribund fish were aseptically dissected to collect spleen samples for bacterial isolation. Tissue homogenates were streaked onto 2216E agar plates (Hopebio, Qingdao, China) and incubated at 30 °C for 24–48 h. Dominant colonies with uniform morphology were sub-cultured on fresh 2216E agar plates to obtain pure isolates for further identification. Genomic DNA was extracted using the TIANGEN Bacterial DNA Kit (TIANGEN Biotech, Beijing, China) following the manufacturer’s instructions, and amplification of the 16S rDNA gene was carried out using the primers listed in Table S1. A PCR was performed in a 25 μL reaction system containing 12.5 μL of 2× Taq PCR Master Mix (Vazyme, Nanjing, China), 1 μL of each primer, 2 μL of template DNA, and 8.5 μL of nuclease-free water. The PCR included an initial denaturation at 95 °C for 5 min followed by 30 cycles of 95 °C for 30 s, annealing at 56 °C for 30 s, extension at 72 °C for 1 min, and a final extension at 72 °C for 7 min. The PCR products were purified and sequenced by Sangon Biotech Co., Ltd. (Sangon, Shanghai, China), sequence alignment was performed using BLAST tool at the National Center for Biotechnology Information (https://blast.ncbi.nlm.nih.gov/Blast.cgi, accessed on 16 November 2025), and phylogenetic trees were constructed using the neighbor-joining method in MEGA 7.0 with 1000 bootstrap replicates [19]. Bacterial characteristics were determined by Gram staining, phenotypic identification was performed using Vibrio-specific biochemical test kits (Hangzhou Microbial Reagent Co., Ltd., Hangzhou, China), and taxonomic classification was based on Bergey’s Manual of Systematic Bacteriology (2nd edition) and Bacteria and Fungi from Fish and Other Aquatic Animals: A Practical Identification Manual [20,21].

### 2.3. Transmission Electron Microscopy

To observe the morphological structure of the bacterial isolates, negative staining and transmission electron microscopy (TEM) were performed. No chemical fixation was performed prior to negative staining. To collect the cells, 1 mL of bacterial culture in the logarithmic growth phase was centrifuged at 10,000× *g* for 10 min at 4 °C. The resulting pellet was washed twice with 0.1 M phosphate-buffered saline (PBS) and resuspended. A drop of this bacterial suspension was placed onto a carbon-coated copper grid and left to stand for 5–10 min, excess liquid was removed with filter paper, and the grid was negatively stained with 2% phosphotungstic acid (pH 7.0) for 1 min. The sample was then blotted dry and subsequently air-dried. TEM observation and image acquisition were carried out using a JEOL JEM-1400 transmission electron microscope (JEOL Ltd., Akishima, Tokyo, Japan) at an accelerating voltage of 80 kV.

### 2.4. Virulence Genetic Testing

For this experiment, eight virulence-related genes were selected in *V*. *harveyi*, including *toxR* and *toxS* (components of a conserved two-component regulatory system), the quorum sensing master regulator *luxR*, the hemolysin genes *vhhA* and *vhhB*, the metalloproteinase genes *vhpA* and *vhpB*, and the zinc metalloproteinase gene *pap6*. These genes were chosen based on their established roles in virulence regulation, tissue damage, and the availability of validated primers for PCR amplification. Specifically, toxR and toxS (components of a conserved two-component regulatory system) and luxR are conserved regulators involved in the expression of virulence factors, while vhhA, vhhB, vhpA, vhpB, and pap6 have been implicated in host tissue damage and pathogenicity in *V*. *harveyi* [22,23,24]. The PCR reaction system was 25 µL and the corresponding primers used for gene amplification are listed in Table S1. The amplification procedure was pre-denaturation at 95 °C for 3 min, denaturation at 95 °C for 15 s, annealing at 50 °C for 15 s, extension at 72 °C for 2 min for 35 cycles, and a final extension at 72 °C for 5 min. The products were visualized via 1% agar gel electrophoresis.

### 2.5. Drug Sensitivity Tests

The antibiotic susceptibility testing of *V. harveyi* was performed using the disk diffusion method on 2216E agar plates, following the Clinical and Laboratory Standards Institute (CLSI) guidelines. The bacterial strain was cultured in 2216E broth at 28 °C with orbital shaking at 180 rpm until the logarithmic growth phase was reached. The bacterial suspension was adjusted to a turbidity equivalent to 0.5 McFarland standard and uniformly spread onto the 2216E agar plates using sterile cotton swabs. Eighteen commercial antibiotic disks (Hangzhou Microbiology Reagent Co., Ltd., Hangzhou, China) including ampicillin (10 μg), ceftriaxone (30 μg), ceftazidime (30 μg), cefotaxime (30 μg), meropenem (10 μg), imipenem (10 μg), gentamicin (10 μg), amikacin (30 μg), streptomycin (10 μg), tetracycline (30 μg), doxycycline (30 μg), chloramphenicol (30 μg), florfenicol (30 μg), ciprofloxacin (5 μg), enrofloxacin (5 μg), norfloxacin (10 μg), trimethoprim-sulfamethoxazole (25 μg), and nitrofurantoin (300 μg) were placed on the agar plates and incubated at 30 °C for 24 h. The diameters of inhibition zones were measured using a digital caliper (mm) and antibacterial susceptibility was interpreted based on the CLSI criteria [25].

### 2.6. Experimental Infections and Sampling

Healthy greater amberjacks (*n* = 20) were randomly assigned into experimental and control groups, with ten fish in each group. *Vibrio harveyi* was cultured in a 2216E medium at 28 °C and 180 rpm until the logarithmic growth phase was reached. The bacterial culture was then centrifuged at 4000× *g* for 10 min at 4 °C, and the resulting pellet was washed twice with sterile PBS. It was then resuspended and adjusted to a final concentration of 1.0 × 10^8^ CFU/mL using PBS. The fish in the experimental group were intraperitoneally injected with 0.1 mL of the bacterial suspension, while those in the control group received 0.1 mL of sterile PBS. After injection, all fish were maintained under identical aquaculture conditions, with a water temperature of 30.10 ± 0.21 °C and a salinity of 30.46 ± 0.26 ppt, and were monitored regularly. At 48 h after injection, spleens from fish in both groups were collected aseptically to prevent contamination. Each sample was halved with one part fixed in 4% paraformaldehyde and the other stored in liquid nitrogen for RNA-Seq analysis.

### 2.7. Histopathological Observations

The fixed spleen samples were processed through a graded ethanol series for dehydration. The tissues were embedded in paraffin and sectioned with a thickness of 4 μm using a rotary microtome and were then stained with hematoxylin and eosin, mounted using neutral balsam, and air-dried. Histopathological changes were observed under a light microscope (Olympus CX23, Olympus Corporation, Tokyo, Japan) at magnifications of 100× and 400× to evaluate tissue damage.

### 2.8. Transcriptome Sequencing and Analysis

Total RNA was extracted using Trizol (Vazyme, Nanjing, China). RNA degradation and DNA contamination were assessed using 1% agarose gel electrophoresis. RNA purity and concentration were measured using a Nanodrop2000 spectrophotometer (Thermo Fisher Scientific, Waltham, MA, USA) and RNA integrity was evaluated by the Agilent 2100 bioanalyzer (Agilent Technologies, Santa Clara, CA, USA). The cDNA libraries were prepared using the Hieff NGS^®^ Ultima dual-mode mRNA Library Prep Kit (Yeasen, Shanghai, China) and the quality was assessed using the DNA 1000 assay kit (Agilent Technologies, Santa Clara, CA, USA). Subsequently, cDNA libraries were generated and sequenced on the Illumina Hiseq 4000 platform by Gene Denovo Biotechnology Co., Ltd. (Guangzhou, China).

Quality control of the raw data was performed using fastp (version 0.18.0). Clean reads were obtained by removing all A bases, reads containing adapters, and those with a N content ratio greater than 10%. These clean reads were then aligned to the rRNA database using Bowtie2 (version 2.2.8) to remove rRNA contamination reads. The clean reads were compared to the fish reference genome using HISAT2 (version 2.1.0), and after alignment, the transcripts were assembled using StringTie (version 1.3.1). The raw sequence data have been submitted to the NCBI Sequence Read Archive (SRA) with accession number PRJNA135468.

### 2.9. Differential Expression and Enrichment Analysis

The expression level of each gene was quantified using HTSeq and expression was presented as the number of fragments per kilobase per million (FPKM). The differentially expressed gene (DEG) analysis was performed using DESeq2, with the screening criteria set with q < 0.05 and log2 fold change (FC) > 1. All genes were functionally annotated by DIAMOND using the gene ontology (GO) knowledge base and the Kyoto Encyclopedia of Genes and Genomes (KEGG) database. Subsequently, an enrichment analysis was performed on the DEGs to identify GO terms and KEGG pathways, which were considered significantly enriched using a threshold of q < 0.05.

### 2.10. Real-Time Quantitative PCR (RT-qPCR) Validation

To validate the reliability of the RNA-Seq data, 17 DEGs were selected for RT-qPCR analysis, including eight up-regulated genes—collectin sub-family member 12 (*colec12*), C1q and tumor necrosis factor related protein 6 (*c1qtnf6*), interleukin 12 (*il-12*), DNA repair protein RAD52 (*rad52*), scavenger receptor cysteine-rich domain-containing protein 5D (*ssc5d*), tumor necrosis factor receptor superfamily member 6b (*tnfrsf6b*), tripartite motif containing 25 (*trim25*), and tumor protein p53 inducible nuclear protein 2 (*tp53inp2*)-and nine down-regulated genes—B-cell CLL/lymphoma 11A (*bcl11a*), C1q and tumor necrosis factor related protein 2 (*c1qtnf2*), cluster of differentiation 79A (cd79a), interferon alpha 3-like (*ifna3*), NLR family CARD domain containing 3 (*nlrc3*), toll-like receptor 7 (*tlr7*), tripartite motif containing 2 (*trim2*), tripartite motif containing 39 (*trim39*), and tumor necrosis factor (*tnf*). β-actin was used as the control. The RT-qPCR was performed on a BIO-RAD CFX96 real-time PCR system using Vazyme SYBR^®^ Green qPCR Master Mix (Vazyme, Nanjing, China). Each 10 μL reaction contained 5 μL of 2× SYBR Green Master Mix, 0.4 μL of each primer, 1 μL of cDNA template, and 3.2 μL nuclease-free water. The cycling conditions were initial denaturation at 95 °C for 3 min followed by 40 cycles of 95 °C for 10 s, annealing at 58 °C for 30 s, and extension at 72 °C for 30 s. A melting curve analysis was performed from 65 °C to 95 °C in 0.5 °C increments every 5 s to verify specificity. The relative expression levels were calculated using the 2^−ΔΔCT^ method. Each sample was run in triplicate to ensure reproducibility.

### 2.11. Data Analysis

All statistical analyses were performed using IBM SPSS Statistics (version 27.0). All data are presented as mean ± SD and all graphs were generated using GraphPad Prism (version 9.0).

## 3. Results

### 3.1. Morphological and Biochemical Characteristics of Bacteria

**Figure 1:**
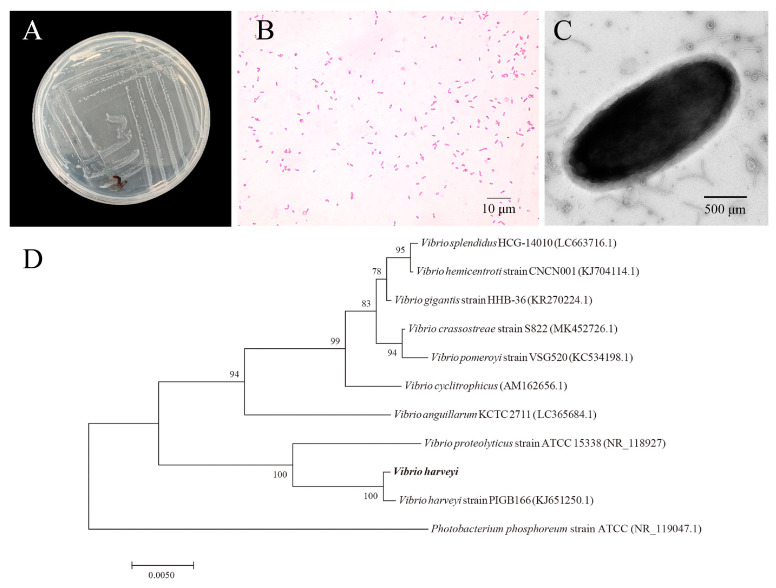
Morphological and phylogenetic characterization of *V. harveyi* strain Vh-2. (**A**) Colony morphology on 2216E agar showing circular, milky-white, semi-translucent colonies with a smooth, moist surface, distinct central elevation, and well-defined boundaries. (**B**) Gram staining revealing Gram-negative, short, rod-shaped cells (scale = 10 μm). (**C**) TEM image revealing short, slightly curved, rod-shaped cells (scale = 500 nm). (**D**) 16S rDNA gene sequence phylogenetic tree with Photobacterium phosphoreum as the outgroup tightly clustering strain Vh-2 with *V. harveyi* strain PIGB166 (bootstrap value = 100%), both of which were distinct from other *Vibrio* species (scale = 0.0050).

For microscopic examination, no parasites were found in the moribund *Seriola dumerili* collected from the aquaculture farm on Donghai Island. The bacteria colonies were circular, milky-white, and semi-translucent with a smooth, moist surface, a distinct central elevation, and well-defined boundaries (Figure 1A). Microscopic observation revealed that the cells were Gram-negative and short rod-shaped (Figure 1B) which was confirmed through the presence of short, slightly curved rod-shaped cells using TEM observation (Figure 1C). Biochemical assays indicated that the bacteria were unable to grow in peptone water without added salt, indicating a requirement for saline water. It exhibited moderate halotolerance, with optimal growth occurring at NaCl concentrations of 3–6%, while growth was inhibited at concentrations above 8%. The strain exhibited positive reactions for mannose utilization, lysine decarboxylase activity, and arginine dihydrolase activity. In contrast, it tested negative for glucose and sucrose fermentation, arabinose and inositol metabolism, Voges-Proskauer test, and indole production using the indocyanine substrate (Table 1).

### 3.2. Sequence Analysis of the 16S rDNA Gene

The 16S rDNA gene sequence of the isolated strain was 1379 bp in length. A BLAST analysis showed that the closest match was *V. harveyi* strain PIGB166 (KJ651250.1) with 99.85% sequence identity. Therefore, the isolate was identified as *V. harveyi* and was designated as Vh-2 in this study. A phylogenetic analysis, with Photobacterium phosphoreum used as the outgroup (Figure 1D), revealed that Vh-2 clustered tightly with *V. harveyi* strain PIGB166 with a bootstrap value of 100%, indicating a high degree of similarity. It was clearly separated from other *Vibrio* spp. including *V. proteolyticus*, *V. anguillarum*, and *V. splendidus*, further supporting the identification as *V. harveyi*.

### 3.3. Detection of Virulence Genes and Drug Sensitivity

**Figure 2:**
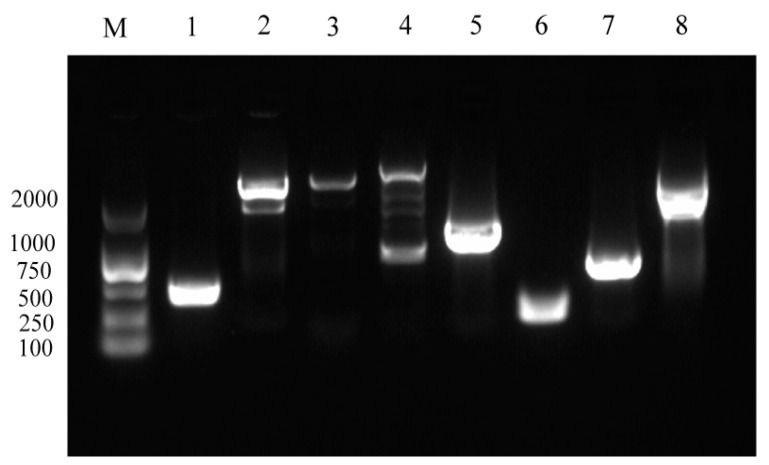
Virulence gene profiling of *V. harveyi* strain Vh-2 by PCR amplification. Lane M: DNA marker, lanes 1–8: amplification products of the eight virulence-associated genes, including tox*R* (1), *toxS* (2), *vhpA* (3), *vhpB* (4), *vhhA* (5), *vhhB* (6), *luxR* (7), and *pap6* (8).

Virulence gene profiling of Vh-2 demonstrated the presence of eight virulence-associated genes, namely *toxR*, *toxS*, *vhpA*, *vhpB*, *vhhA*, *vhhB*, *luxR*, and *pap6* (Figure 2). A total of 29 antibiotics were tested for their activity against the *V. harveyi* strain Vh-2 (Table 2). The strain exhibited susceptibility (S) to ceftriaxone, florfenicol, and meropenem. Intermediate (I) was observed to cefepime, cefoxitin, azithromycin, chloramphenicol, nitrofurantoin, levofloxacin, and ofloxacin. Conversely, the strain was resistant (R) to the remaining 19 antibiotics, including ampicillin, amoxicillin, piperacillin, ampicillin/sulbactam, ceftazidime, erythromycin, norfloxacin, enrofloxacin, ciprofloxacin, metronidazole, streptomycin, kanamycin, gentamicin, amikacin, tetracycline, vancomycin, polymyxins, trimethoprim-sulfamethoxazole, and imipenem.

### 3.4. Histopathological Analyses

**Figure 3:**
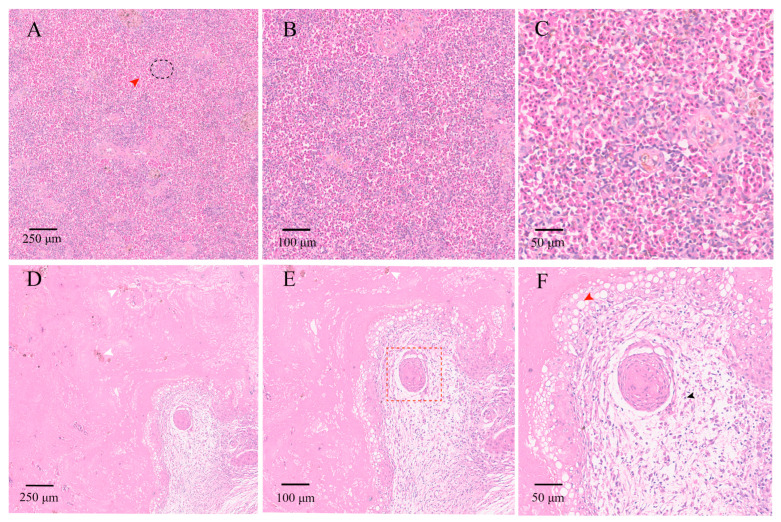
Histological changes in the spleen of *S. dumerili* (H&E staining). (**A**–**C**) Control group: (**A**) 100× (scale = 250 μm), intact spleen structure with clearly defined red and white pulp, Red arrow indicates red pulp; black circle highlights white pulp, (**B**) 200× (scale = 100 μm), normal lymphoid follicles and uniform tissue staining, (**C**) 400× (scale = 50 μm), splenocytes with intact, round nuclei and no pathological alterations. (**D**–**F**) Experimental group: (**D**) 100× (scale = 250 μm), diffuse necrotic areas with pigment deposition (white arrowhead), (**E**) 200× (scale = 100 μm), disrupted tissue architecture, extensive necrosis, and pigment accumulation (white arrowhead), red frame highlights granulomatous lesions, (**F**) 400× (scale = 50 μm), perivascular lymphocyte infiltration (black arrowhead) and marked cytoplasmic vacuolation (red arrowhead), with nuclear fragmentation.

In the control group, the architecture of the spleens was intact with clearly defined red and white pulp and uniform staining. No congestion, hemorrhage, or inflammatory infiltration was observed. Observation under a 100× showed that white pulp was evenly distributed (Figure 3A) and observation under a 200× indicated normal lymphoid follicles (Figure 3B). The splenocytes had intact, round nuclei without vacuolation or degeneration when observed under a 400× (Figure 3C). In the experimental group, extensive lesions were evident on the spleen samples. The diffuse necrosis and pigment deposition (white arrowhead) were visible under a 100× (Figure 3D) and marked structural disorganization, cell loss, and pigment accumulation (white arrowhead) were observed under a 200× (Figure 3E). Furthermore, perivascular lymphocyte infiltration (black arrowhead), prominent cytoplasmic vacuolation (red arrowhead), and nuclear fragmentation were observed under a 400× (Figure 3F).

### 3.5. Transcriptome Sequencing and Assembly

The quantity of raw reads obtained for all samples ranged from 60.22 to 67.53 million, which decreased to 59.64 to 62.68 million clean reads for the control group (CS1–CS4) and 58.43 to 66.97 million clean reads for the experimental group (PS1–PS4) after quality filtering (Table 3). The Q20 values for all samples were up to 97.75%, the Q30 values were more than 93.58%, and the GC content was stable within the range of 47.76–48.43%, indicative of high-quality sequencing data. The construction of the transcriptome was, therefore, successful and suitable for subsequent analysis.

### 3.6. Identification of DEGs and Enrichment Analysis

**Figure 4:**
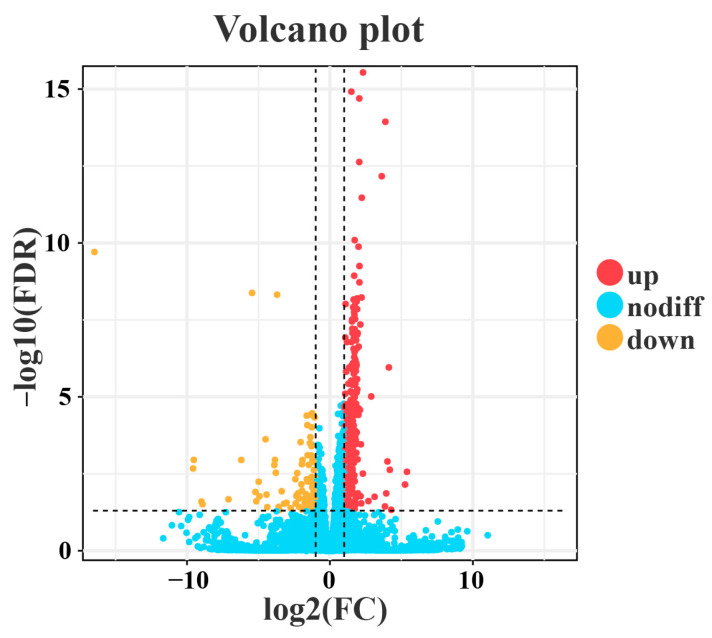
Volcano plot of DEGs between naturally infected and healthy *S. dumerili*. Blue dots represent genes with no significant differential expression, while red and yellow dots denote significantly upregulated and downregulated genes, respectively. The *x*-axis shows the log_2_ fold-change, and the y-axis shows −log10 of statistical significance (q-value). Vertical dashed lines indicate the fold-change threshold (|log2FC| > 1), and the horizontal dashed line indicates the significance threshold (q-value < 0.05).

**Figure 5:**
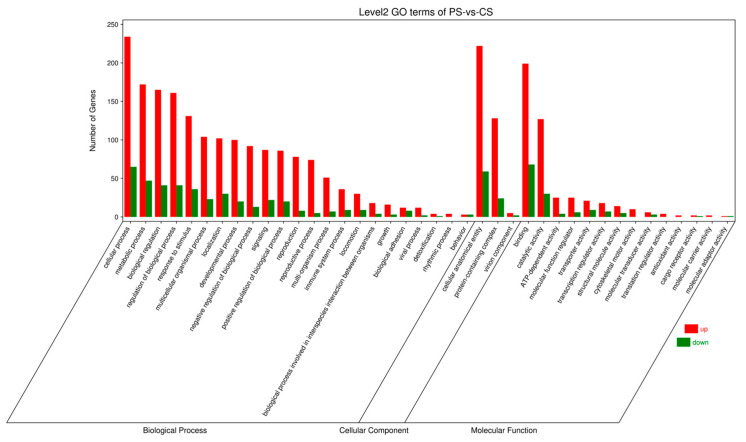
Level 2 Gene Ontology (GO) terms enriched in PS vs. CS. Red and green bars represent the number of up-regulated and down-regulated differentially expressed genes (DEGs), respectively.

**Figure 6:**
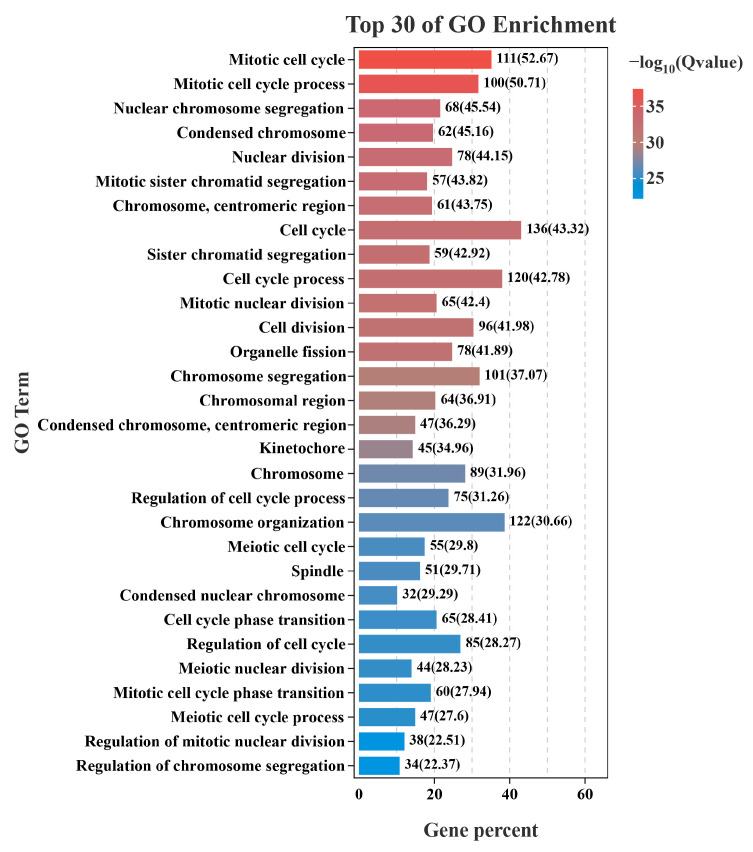
Top 30 enriched GO terms of DEGs ranked by the proportion of annotated genes in *S. dumerili*. The x-axis represents the percentage of DEGs annotated to each GO term (gene ratio). The y-axis lists the GO terms ranked by this percentage. Bar length indicates the gene ratio, and bar color represents the significance of enrichment (−log10(q-value)).

**Figure 7:**
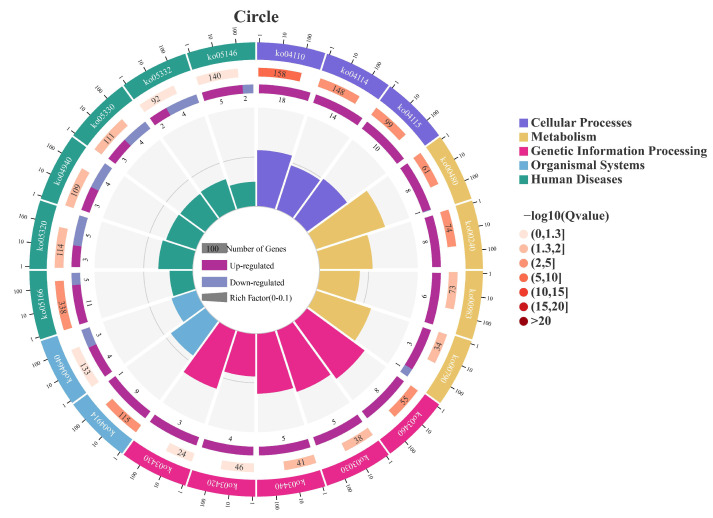
Circular representation of KEGG pathway enrichment in PS vs. CS. Outer circle: the top 20 enriched pathways with a scale indicating the number of DEGs. Second circle: the number of DEGs in the background gene set for each pathway (longer bars indicate higher counts) and q-value (darker red indicates smaller q-value). Third circle: proportion of up-regulated (deep purple) and down-regulated (light purple) DEGs in each pathway. Inner circle: Rich Factor (number of DEGs in the pathway divided by total genes in the pathway), with grid lines at 0.1 intervals.

**Figure 8:**
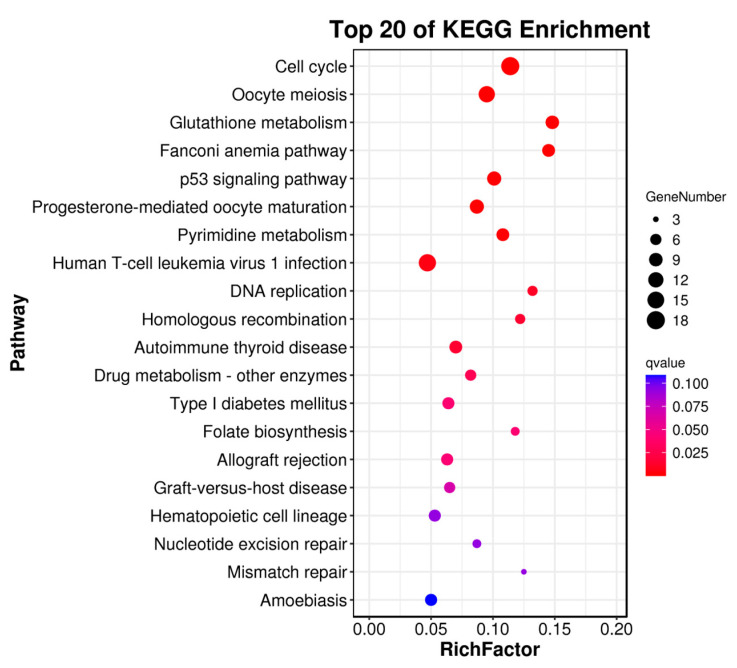
Top 20 significantly enriched KEGG pathways of DEGs in *S. dumerili*. The x-axis represents the gene ratio (proportion of DEGs mapped to each pathway), the *y*-axis lists the KEGG pathway names, bubble size indicates the number of DEGs in each pathway, and bubble color represents the significance of enrichment (−log10 (q-value)).

Through RNA sequencing, a total of 396 DEGs, including 293 upregulated genes and 103 downregulated genes, were obtained (Figure 4). Among these, the top 10 up- and down-regulated DEGs are displayed in Table S2. Subsequently, the GO and KEGG pathways were enriched and analyzed. The GO enrichment analysis assigned the DEGs to 1112 functional terms, including 845 terms related to biological processes, 146 terms to cellular components, and 121 terms to molecules functions (Figure 5). Significantly enriched biological process terms included mitotic cell cycle (GO:0000278), mitotic cell cycle process (GO:1903047), nuclear chromosome segregation (GO:0098813), and nuclear division (GO:0000280). Furthermore, during cellular processes, DEGs were predominantly enriched in condensed chromosome (GO:0000793), chromosome, centromeric region (GO:0000775), chromosomal region (GO:0098687), and condensed chromosome, centromeric region (GO:0000779). In the context of molecular processes microtubule binding (GO:0008017), tubulin binding (GO:0015631), ATP binding (GO:0005524), and adenyl nucleotide binding (GO:0030554) were particularly salient (Figure 6). After functional annotation of DEGs, a total of 150 genes were annotated in the KEGG database, mainly involved in cell cycle regulation, DNA repair, metabolic processes, and immune responses (Figure 7). KEGG enrichment analysis revealed that these genes were predominantly enriched in the Cell cycle, Oocyte meiosis, Glutathione metabolism, Fanconi anemia pathway, and p53 signaling pathway (Figure 8).

### 3.7. Verification of DEGs by RT-qPCR

**Figure 9:**
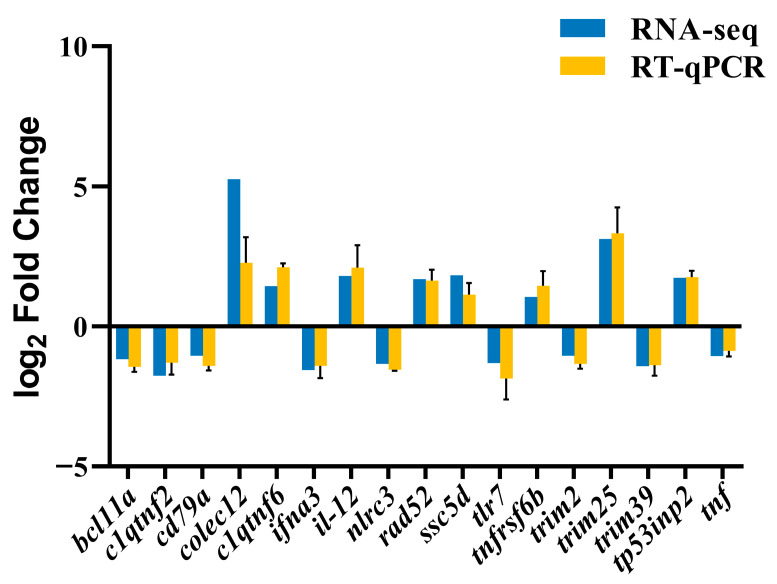
Validation and comparison of candidate gene expression levels by comparing the RNA-Seq and RT-qPCR results. The x-axis shows the gene names, and the y-axis represents the log2 fold change in expression, where the blue and yellow bars (with SD) indicate RNA-Seq and RT-qPCR data, respectively.

To validate the reliability of the RNA-Seq data, the transcriptional levels of 17 selected DEGs were detected by RT-qPCR. These genes covered functional pathways such as immune recognition, signal transduction, inflammatory regulation, and cell repair. The RT-qPCR validation results were highly consistent with the RNA-Seq data, thus confirming the reliability of the transcriptomics sequencing data (Figure 9).

## 4. Discussion

Greater amberjack aquaculture farms have recently been affected by frequent outbreaks of bacterial diseases, with *V. harveyi* being the predominant pathogen. To investigate the pathogenic mechanism of *V. harveyi* and the immune response mechanism of greater amberjack to *V. harveyi*, we characterized the pathogenic features of the *V. harveyi* Vh-2 strain and explored the transcriptomic host-disease responses of the greater amberjack to *V. harveyi* infection. Many DEGs were identified, predominantly involved in immune defense mechanisms, inflammatory responses, and cellular apoptosis.

Through physiological and biochemical characterization and 16S rDNA sequence comparison, the strain isolated from diseased greater amberjack spleen tissue was identified as *V. harveyi*, which has been widely reported as a primary pathogen in marine fish species [26]. The Vh-2 strain exhibited typical physiological and biochemical halophilic characteristics, growing well under 3% and 6% NaCl conditions, but unable to grow in a salt-free environment, demonstrating its salt dependence as a marine halophilic bacterium [27]. Additionally, the strain exhibited positive reactions with lysine and arginine decarboxylase, indicating its capacity for amino acid metabolism, corresponding to the general characteristics of the *Vibrio* genus [28]. The isolated strain failed to ferment glucose, sucrose, arabinose, and inositol, but tested positive for mannose, reflecting individual variability in carbon source utilization [29]. Additionally, *Vibrio* variants may have unstable or even completely negative sugar fermentation capabilities, which may be related to their adaptability to various hosts and environments [30]. The antimicrobial susceptibility patterns of Vh-2 generally align with β-lactam and macrolide resistance and florfenicol and meropenem susceptibility of *V. harveyi* from southern China [31]. Such patterns may be shaped by regional antibiotic practices or strain-specific resistance gene mutations [32,33,34]. This multi-drug resistance phenotype is consistent with trends reported in isolates from diverse geographic regions, which often retain common resistance to protein-synthesis inhibitors despite genotypic variations [30].

The virulence factors of *V. harveyi* have been classified and divided into two categories: widely conserved typical genes (*luxR*, *toxR*, *vhpA*, *chiA*, *SP*, *vhh*, *vhml* and *vhs*) and atypical genes with variable distribution (*zot*, *toxRVc*, *tcpA*, *ctxA*, *hlyA*, *flaC*, *tdh*, *trh* and *vvh*) [35]. In addition to some typical virulence genes (*toxR*, *luxR*, *vhh*, *vhp*), our study also identified *toxS* and *pap6*. Previous studies have demonstrated that *toxS* functions alongside *toxR*, stabilizing it and preventing degradation, thereby ensuring proper regulation of virulence gene expression [36]. Althuohg we did not perform sequencing analysis on the virulence gene amplicons, previous literature indicates that the selected core regulatory genes (e.g., *toxR* and *luxR*) are highly conserved across *V. harveyi* and *Harveyi* clade strains; the hemolysin genes *vhhA*/*vhhB* may exhibit copy duplication in highly virulent strains (with 98.8% homology), whereas the metalloproteases *vhpA*/*vhpB*/*pap6* share conserved structural domains, occasionally resulting in non-specific amplification products during PCR (as observed in the vhpB lane), and no significant SNPs leading to amino acid changes have been reported [22,35,37]. Specifically, the *pap6* gene, originally cloned from *V. harveyi* AP6, encodes a zinc-dependent metalloprotease capable of degrading gelatin, fibronectin, and type IV collagen, suggesting its potential role in host tissue damage and infection [37]. Histopathology aids in assessing structural damage to organs post-infection and findings have revealed significant structural damage to greater amberjack spleen (extensive cellular necrosis, red blood cell exudation, and increased inflammatory cells) infected with *V. harveyi*. This is consistent with histopathological splenic lesions reported in other fish species, such as groupers and sea bass, infected with *Vibrio* spp., which often displayed red blood cell extravasation and lymphocyte aggregation, hallmarks of the spleen’s dual role in blood filtration and inflammatory regulation [38,39].

Innate immunity constitutes the first line of defense against microbial infections [40], which recognizes various pathogen-associated molecular patterns via specific pattern-recognition receptors such as Toll-like receptors (TLR), RIG-I-like receptors (RLR), and NOD-like receptors (NLR) [41]. Enrichment of TLR, RLR, and NLR pathways, along with differential expression of *tlr7* (downregulated), *trim25* (upregulated), and *nlrc3* (downregulated), indicates that pathogen recognition and subsequent innate immune signaling were actively modulated in *S. dumerili* following *V. harveyi* infection. The endosomal pattern-recognition receptor *tlr7* detects single-stranded RNA and activates MyD88-IRF7-dependent type I interferon (IFN) production [42]. In this study, we found that *tlr7* was downregulated in *S. dumerili* in response to *V. harveyi* infection, similar to observations in vivo during porcine epidemic diarrhea virus infection where *tlr7* downregulation resulted in the inhibition of NF-kappa B signaling and reduced TNF-α expression. It was recently reported that TLR7 expression is downregulated in the livers of patients infected with hepatitis C, which may be associated with immune negative feedback mechanisms or virus-mediated immune evasion [43]. In contrast, TRIM25 acts as a key E3 ubiquitin ligase that mediates K63-linked ubiquitination of RIG-I, enhancing antiviral signaling [44]. It has been suggested that common carp (*Cyprinus carpio*) RIG-I and TRIM25 effectively inhibit spring viremia of carp virus replication by inducing phosphorylation of TBK1, IRF3, and p65 [45]. Similarly, during nervous necrosis virus infection in red spotted grouper (*Epinephelus akaara*), zebrafish TRIM25 acted as an enhancer of the RLR signaling pathway [46]. Our results suggest that the RLR signaling pathway in S. dumerili may play an important role in host immune defense against *V. harveyi*. As *nlrc3* expression was downregulated in *S. dumerili* after *V. harveyi* infection, this suggests a release of negative immune regulation. The negative regulatory role of NLRC3-like may provide valuable insights into how homeostasis is maintained and the causes of autoimmune and inflammatory diseases [47]. In teleosts, NLRC3-like proteins can inhibit NOD1-RIP2 or STING pathways, exerting both suppressive and activating effects on the regulation of pathogen-induced inflammation [48,49]. Downregulation of *nlrc3* may relieve this inhibition, enhancing pro-inflammatory responses, promoting bacterial elimination, and limiting pathogen spread [50]. These changes suggest that an innate immune response played an essential role in great amberjacks when infected with *V. harveyi*.

Transcriptomic analysis revealed pronounced upregulation of pro-inflammatory cytokines, notably *il-12* and *tnf-α*. Cytokine–cytokine receptor interactions and JAK–STAT pathways were significantly enriched, with *il-12* and *tnf-α* showing notable expression changes. While essential for recruiting immune cells to the infection site and eliminating bacteria [51,52], cytokines from the IL-12 family are recognized for their critical roles in cancer, infections, and inflammatory conditions by regulating both innate and adaptive immunity [53]. IL-12, comprising IL-12p35 and IL-12p40, is a multifunctional cytokine involved in inflammation regulation and the bacterial immune response [54]. *Seriola dumerili* il-12 was upregulated after *V. harveyi* infection, consistent with its role in promoting Th1 polarization and macrophage activation in teleosts [55]. LPS, polyI:C, IFN-γ, and IL-1β can trigger IL-12 production in rainbow trout (*Oncorhynchus mykiss*) macrophages [56]. Recombinant IL-12 has been shown to stimulate peripheral blood lymphocyte proliferation and induce *tnf-α* expression in groupers, suggesting a conserved role in pro-inflammatory and cell-mediated immunity [57]. As a central mediator of inflammatory responses, *tnf-α* in fish have shown increased expression during the initial phases of infection [58]. In turbot (*Scophthalmus maximus*) infected with *Aeromonas salmonicida* and rainbow trout infected with *Yersinia ruckeri*, there were significant increases in tnf-α transcripts [59,60,61]. Conversely, our study shows that tnf-α expression in *S. dumerili* was downregulated after *V. harveyi* infection. Moreover, gene expression kinetics in flounder were studied using a challenge model that simulated both acute and chronic *Nocardia seriolae* infections, revealing a significant increase in tnf-α during the early stages of acute infection, followed by an unexpected downregulation [62]. Based on this, we can assume that the *V. harveyi* infection in the great amberjack was chronic, which may represent a regulatory counterbalance to prevent excessive tissue damage during systemic infection.

It is important to note the limitations of our study. The single time-point analysis only captures a snapshot of the dynamic host–pathogen interactions. Future studies should incorporate a time-series analysis to delineate the kinetic progression of the immune responses in fish. Furthermore, the functional role of the candidate genes identified here, such as il-12, requires validation through in vivo functional assays.

## 5. Conclusions

In conclusion, this study systematically described the pathogenic mechanism of *V. harveyi* and screened the primary DEGs and pathways involved in pathogen recognition, inflammation, and immune response to *V. harveyi* infection in the greater amberjack. These findings have improved our knowledge of the immune responses of greater amberjack to *V. harveyi* and will assist in producing effective vaccines, treatments, and disease-resistant strains.

## Figures and Tables

**Table 1:** Physiological and biochemical characteristics of *V. harveyi* strain Vh-2.

Characteristics	Vh-2	*Vibrio harveyi*
1% NaCl glucose	−	−
1% NaCl dextran peptone water (VP)	−	−
1% NaCl peptone water (indigo matrix)	−	+
1% NaCl sucrose	−	−
1% NaC1 mannose	+	+
1% NaCl arabinose	−	−
1% NaCl Inositol	−	−
1% NaC1 lysine	+	+
1% NaCl amino acid control	+	−
1% NaCl arginine dihydrolase	+	−
1% NaCl arginine dihydrolase control	−	−
Unsalted peptone water	−	−
3% NaCl peptone water	+	+
6% NaC1 peptone water	+	+
8% NaC1 peptone water	−	+
10% NaC1 peptone water	−	−
Gram staining	G−	G−

Abbreviations: +, positive; −, negative; G− = Gram-negative.

**Table 2:** Results of the antimicrobial susceptibility tests of *V. harveyi* strain Vh-2.

Group	Name	Drug Concentration (μg Per Disk)	Bacteriostatic Ring Diameter (mm)	Sensitivity
β-Lactams	Ampicillin	10	6.67	R
	Amoxicillin	20	8.89	R
	Piperacillin	100	0	R
	Ampicillin/ Sulbactam	10/10	11.80	R
	Cefepime	30	20.35	I
	Ceftriaxone	30	24.63	S
	Cefoxitin	30	16.28	I
	Ceftazidime	30	11.09	R
Macrolides	Erythromycin	15	7.62	R
	Azithromycin	15	6.77	R
Quinolones	Norfloxacin	10	13.09	I
	Enrofloxacin	10	11.67	R
	Ciprofloxacin	5	13.03	R
Nitroimidazoles	Metronidazole	5	8.89	R
Aminoglycosides	Streptomycin	10	10.08	R
	Kanamycin	30	7.64	R
	Gentamicin	10	10.65	R
	Amikacin	30	10.92	R
Tetracyclines	Tetracycline	30	8.94	R
Amphenicols	Chloramphenicol	30	14.08	I
	Florfenicol	30	22.45	S
Nitrofurans	Nitrofurantoin	300	16.40	I
Glycopeptides	Vancomycin	30	6.84	R
Polypeptides	Polymyxins	300	7.51	R
Sulfonamides	Trimethoprim- sulfamethoxazole	23.75/1.25	7.50	R
Carbapenems	Meropenem	10	25.59	S
	Imipenem	10	18.28	R
Fluoroquinolones	Levofloxacin	5	14.59	I
	Ofloxacin	5	14.74	I

Note: S: susceptible; I: intermediate; R: resistant.

**Table 3:** Summary of sequencing data and quality control for the RNA-Seq samples.

Sample	Raw Reads	Clean Reads	Q20 (%)	Q30 (%)	GC Content (%)
CS1	6021776400	5964040893	98.05	94.43	48.06
CS2	6459607800	6399469682	97.87	93.96	48.13
CS3	6233102400	6268793066	98.00	94.37	48.43
CS4	6075345000	6025193963	97.93	94.11	48.09
PS1	6753404700	6696650167	97.76	93.61	48.23
PS2	6201612300	6144420725	97.75	93.58	47.98
PS3	5892544800	5843028687	98.07	94.38	48.07
PS4	5934583500	5878948379	97.93	94.07	47.76

## Data Availability

The data that support the findings of this study are available upon reasonable request. The raw reads used in this article have been deposited into the Sequence Read Archive (SRA) of the NCBI database under BioProject accession number: PRJNA135468.

## Supplementary Materials

The following supporting information can be downloaded at: https://www.mdpi.com/article/10.3390/ani16020184/s1, Table S1: Primers sequences used in this study; Table S2: Top ten up and down-regulated DEGs in the spleen of *Seriola dumerili* following *Vibrio harveyi* infection.
