# Supplementary material for: Pathogenic Characterization and Host Immune Response to Vibrio harveyi in Diseased Seriola dumerili
Source: Animals (Basel). 2026 Jan 8;16(2):184. doi: 10.3390/ani16020184 (PMC12837687; doi:10.3390/ani16020184)
Supplement: Supplementary file 1 [file animals-16-00184-s001.zip › Table S1.pdf]

Table S1. Primers sequences used in this study.

Gene name	Primers sequence (5' - 3')	Usage
toxR	F:TTCTGAAGCAGCACTCAC	Virulence Detection
	R:TCGACTGGTGAAGACTCA	
toxS	F:TCCGTTCTTTTCAGCAGTTG	
	R:GCTGTGCTTCGGAAAAGTCT	
vhpA	F:TGAACGACGCCCATTATT	
	R:CCATTCGGGAACTTGTTACC	
vhpB	F:GCGATGCCGACGACAAT	
	R:CTGCCCTGCTTCCTGTGT	
vhhA	F:ATCATGAATAAAACTATTACGTTACT	
	R:GAAAGGATGGTTTGACAAT	
vhhB	F:TCAGTGCCTCTCAAGTAAGA	
	R:GCTTGATAACACTTTGCGGT	
luxR	F:ATCTTGCGGCGTGTAGTG	
	R:AGTTGGTTAGTGCGGTTTGT	
pap6	F:ACTGTGAAAGCGGTCAAC	
	R:GCAAGGTAGGCGTCAGAG	
16s	27F:AGAGTTTGATCATGCTCAG	16s rDNA
	1492R:GGTACCTTGTGTACGACTT	
rad52	F:CTGAGTATTTGTCAACGCTT	RT-qPCR
	R:CATCACAGACATAGCACCA	amplification
ifna3	F:TCCCGTCATGTTCTCGAAG	
	R:GTCAACTCTCATTAGATGGC	
c1qtnf6	F:AAGATCTCGTGTTTTGGACT	
	R:CCGTCTGTTGTTTAGCACGAA	
ssc5d	F:CATCATTAAGTTGTCGCTGT	
	AGCCAATTGAGATTGTCGTC	
colec12	F:GAGCCAGACAACAACCAGT	
	R:TACAGGGCACATCGAACCAG	

tnfrsf6b	F:ATGCTCCTCTTTCCGTTGCT
	R:ACAGGTGAGACTATCCCCAT
tp53inp2	F:GCCACCAGTCAAGCCATCTCT
	R:GCTGTTGGAGGTTGAAGGAGTG
cd79a	F:ACAAAATCGGTCCAGTCAC
	R:GCCCAGTGTCATTCAGC
tlr7	F:TGTTCCTTCTTGGCTTTGACA
	R:CCAAAGCACCTGTAAGCAA
il-12	F:TTCTTTGGCCTGCAGTTCCA
	R:CTTGGCTTTGACTGTAACCTC
nlrc3	F:ACCTGCAAGAGATGAACCA
	R:TTGTCTTCAGCCCAGTCCA
bcl11a	F:GGAAGCACCACTACCGTCA
	R:CACCTTGATGCGCTTGGACA
c1qtnf2	F:AGTTTGTCTGCGCTATACCTG
	R:TGTACTGTCCATTTTGCACCA
tnf	F:AGATCCCCACTACACGCTGA
	R:CCACTTTCGAGCTCTTGTCC
trim39	F:CCTTGCACATATCTATGGCAG
	R:CATCTCAGGGCAAATGTCA
trim2	F:CGCTGAAGAAAACAACCGAGA
	R:GACTCCTCAAAGCACCAGT
trim25	F:AAACATTTGAAGCGGCACT
	R: AGCCAGACCTTCAATGGAAC
