# Supplementary material for: Pathogenic Characterization and Host Immune Response to Vibrio harveyi in Diseased Seriola dumerili
Source: Animals (Basel). 2026 Jan 8;16(2):184. doi: 10.3390/ani16020184 (PMC12837687; doi:10.3390/ani16020184)
Supplement: Supplementary file 1 [file animals-16-00184-s001.zip › Table S2.pdf]

Supplementary Table S2. Top ten up and down-regulated DEGs in the spleen of *Seriola dumerili* following *Vibrio harveyi* infection.

Down Gene ID	Symbol	log2(fc)	Up Gene ID	Symbol	log2(fc)
ncbi_111224369	*DNASE1*	2.898336344	MSTRG.4465	--	-16.48136
ncbi_111226613	*mxd3*	1.219489238	ncbi_111240397	*APOB*	-5.450294
ncbi_111220888	*anln*	2.021400258	ncbi_111223274	--	-3.701366
ncbi_111232691	--	1.157655406	ncbi_111236727	*RAVER2*	-1.238988
ncbi_111234421	*KIF20A*	2.350616901	MSTRG.9968	*IGLC1*	-1.348920
ncbi_111218441	*CPXV209*	6.802020828	ncbi_111216460	*CCN2*	-1.626377
ncbi_111234089	*IQGAP3*	3.400408931	ncbi_111236831	*Pik3ca*	-1.068107
ncbi_111237114	*nusap1*	8.12773516	MSTRG.20186	*PIGR*	-1.577244
ncbi_111221856	*CIT*	1.329410794	MSTRG.22232	*Btn2a2*	-1.274205
ncbi_111233225	*donson*	5.650173711	ncbi_111224495	*CACNA2D4*	-1.365761
